# Altered Plasma Membrane Lipid Composition in Hypertensive Neutrophils Impacts Epithelial Sodium Channel (ENaC) Endocytosis

**DOI:** 10.3390/ijms25094939

**Published:** 2024-04-30

**Authors:** Yolanda Ríos-Medina, Pedro Rico-Chávez, Ivette Martínez-Vieyra, Juan C. Durán-Álvarez, Mario Rodriguez-Varela, Ruth Rincón-Heredia, César Reyes-López, Doris Cerecedo

**Affiliations:** 1 Laboratorio de Hematobiología, Escuela Nacional de Medicina y Homeopatía, Instituto Politécnico Nacional, Mexico City 07230, Mexico; yriosm1800@alumno.ipn.mx (Y.R.-M.); pricoc1100@alumno.ipn.mx (P.R.-C.); iamartinez@ipn.mx (I.M.-V.); 2 Instituto de Ciencias Aplicadas y Tecnología, Universidad Nacional Autónoma de México, Mexico City 04510, Mexico; carlos.duran@icat.unam.mx (J.C.D.-Á.); mario.rodriguez@icat.unam.mx (M.R.-V.); 3 Instituto de Fisiología Celular, Universidad Nacional Autónoma de México, Mexico City 04510, Mexico; rrincon@ifc.unam.mx; 4 Laboratorio de Bioquímica Estructural, Escuela Nacional de Medicina y Homeopatía, Instituto Politécnico Nacional, Mexico City 07230, Mexico; careyes@ipn.mx

**Keywords:** ENaC regulatory complex, neutrophils, membrane lipids, proteasome, endosome degradation

## Abstract

Biological membranes are composed of a lipid bilayer with embedded proteins, including ion channels like the epithelial sodium channel (ENaC), which are critical for sodium homeostasis and implicated in arterial hypertension (HTN). Changes in the lipid composition of the plasma membrane can significantly impact cellular processes related to physiological functions. We hypothesized that the observed overexpression of ENaC in neutrophils from HTN patients might result from alterations in the structuring domains within the plasma membrane, disrupting the endocytic processes responsible for ENaC retrieval. This study assessed the structural lipid composition of neutrophil plasma membranes from HTN patients along with the expression patterns of key elements regulating ENaC at the plasma membrane. Our findings suggest alterations in microdomain structure and SGK1 kinase activity, which could prolong ENaC presence on the plasma membrane. Additionally, we propose that the proteasomal and lysosomal degradation pathways are insufficient to diminish ENaC presence at the plasma membrane in HTN. These results highlight the importance of understanding ENaC retrieval mechanisms and suggest that targeting these mechanisms could provide insights for developing drugs to prevent and treat HTN.

## 1. Introduction

Cell membranes are primarily composed of fatty acid-based lipids and proteins. Phosphatidylcholine (PC), phosphatidylethanolamine (PE), and phosphatidylserine (PS) are the main glycerol backbone-containing phospholipids found in most organelle membranes [1]. Phosphatidylcholine (PC), as the most abundant phospholipid in organelles, plays a key role in organizing planar bilayers due to its cylindrical shape. In contrast, phosphatidylethanolamine (PtdEtn), the second most abundant phospholipid, can adopt a hexagonal phase, which is a unique characteristic among glycerophospholipids [2]. The presence of phosphatidylethanolamine (PE) alongside phosphatidylcholine (PC) facilitates the generation of spontaneous curvature, which is necessary for supporting fission and fusion processes in vesicular transport [3]. Both phospholipids serve as substrates for the enzymatic production of PS through base exchange reactions [4]. Although PS is a minor component of eukaryotic membranes, it contributes a negative surface charge to them [5]. Sphingolipids represent another critical component of the plasma membrane’s external leaflet, displaying a diverse range of headgroup sizes, including hydroxyl groups (ceramide), phosphocholine (sphingomyelin, SM), or complex sugar-modified headgroups collectively referred to as glycosphingolipids [6]. Cholesterol, the third essential class of lipid molecules in mammalian cells, modulates membrane fluidity and permeability by interacting with neighboring lipids such as PC and SM [7,8]. Moreover, cholesterol regulates the activity of various membrane-bound proteins, enzymes, ion channels, receptor-associated protein kinases, and sensor proteins [9].

The specific composition of membrane lipids plays a crucial role in shaping the lipid environment surrounding the epithelial sodium channel (ENaC), thereby influencing its function and endosomal recycling pathway [10]. ENaC plays a crucial role in maintaining sodium homeostasis, regulating extracellular fluid volume, and controlling blood pressure [11]. Its significance is especially notable in the context of hypertension (HTN), a serious and multifactorial medical condition influenced by various factors such as genetics, environment, demographics, lifestyle, and vascular and neuroendocrine disorders [12]. ENaC is predominantly expressed at the apical membranes of various tissues, including the distal nephron’s principal cells in the aldosterone-sensitive distal nephron (ASDN) and the collecting duct, as well as in non-renal tissues like the endothelium, vascular smooth muscle, tongue, and colon [13,14,15]. ENaC functions as a heterotrimeric channel comprising α, β, and γ subunits, exhibiting high selectivity for sodium ions [16]. Notably, cholesterol levels play a significant role in modulating ENaC activity. Reduction in cholesterol levels leads to a decrease in both the open probability [17] and the surface expression of ENaC channels [18]. Furthermore, cholesterol interacts with cholesterol-rich membrane domains, commonly known as lipid rafts, to modulate ion channels. This association adds complexity to ion channel regulation, as channels within lipid rafts can directly bind to cholesterol and interact with multiple signaling molecules segregated within these specific raft domains.

The most potent downregulation of ENaC occurs through an intricate interplay among ENaC, caveolin-1 (Cav-1), and Nedd4-2 and plays a pivotal role in the internalization and ubiquitination of ENaC, ultimately influencing its activity and abundance on the surface of epithelial cells [19]. Caveolin-1 negatively regulates this process, thereby impacting the surface expression of ENaC. Additionally, it has been observed that the activation of ENaC by SGK1 may necessitate the presence of an intact lipid environment and/or lipid rafts as a signaling platform [20].

In response to ubiquitination, a protein is internalized through the endosomal system and trafficked to multivesicular bodies for degradation in lysosomes [21]. This process occurs regardless of the mode of endocytosis, whether it is clathrin- and caveolae-independent or clathrin- and caveolae-mediated endocytosis. (16). The subunits of ENaC (α, β, γ) are either polyubiquitinated, monoubiquitinated, or multimonoubiquitinated at the cell surface [22] and are targeted to endosomal/lysosomal degradation pathways [23]. The ENaC subunits that are not directed to the plasma membrane are likely to be polyubiquitinated and degraded through the proteasomal pathway.

Recently, our research findings have shown that the ENaC is upregulated in neutrophils from patients with hypertension (HTN), which exhibit characteristic biochemical and morphological features indicative of an activated state, including elevated expression of caveolin and abnormal fluidity of the plasma membrane [24].

Liquid chromatography tandem mass spectrometry analyses provided insights into the cholesterol and structural phospholipid composition imbalance within the plasma membrane of neutrophils sourced from individuals with hypertension, which might potentially affect the internalization of α-ENaC and its retention within the membrane of hypertensive individuals. Through Western blotting and confocal analysis assays, we determined the presence of some proteins that contribute to the ubiquitination process of α-ENaC—scrutinizing its degradation within proteosomes or lysosomes—which apparently are insufficient to retrieve α-ENaC from the plasma membrane of neutrophils from HTN patients.

## 2. Results

### 2.1. Imbalance of the Phospholipid and Cholesterol Composition at the Plasma Membrane of Neutrophils Derived from Hypertensive (HTN) Patients

**Figure 1:**
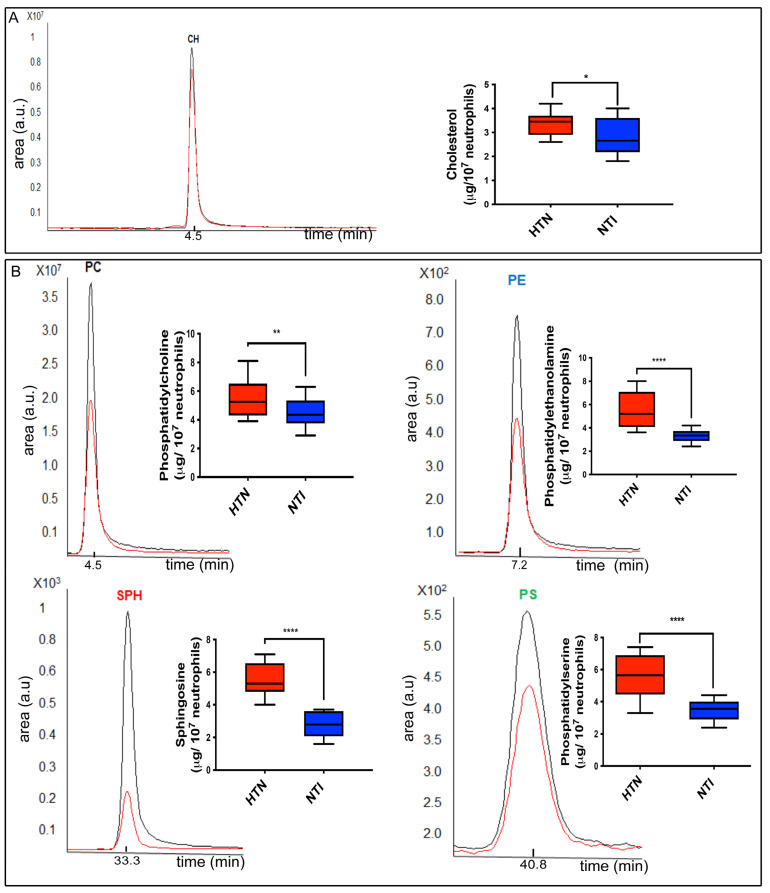
Changes in the lipidic composition of neutrophils from patients with HTN. (**A**) LC-MS/MS for cholesterol (red line, HTN neutrophils; black line, NTI neutrophils) and graphical representation of the concentration of cholesterol in the lipid extracts of neutrophils from patients with HTN (red) and NTI (blue). (**B**) LC-MS/MS for cholesterol (red line, HTN neutrophils; black line, NTI neutrophils) and graphical representation of the concentration of cholesterol in the lipid extracts of neutrophils from patients with HTN (red) and NTI for phosphatidylcholine (PC), phosphatidylethanolamine (PE), sphingosine (SPH), and phosphatidylserine (PS). Values are mean ± standard error (SE) for three biological replicates from six individuals in each group; * *p* = 0.011, ** *p* = 0.012, **** *p* = 0.0001; unpaired *t*-test.

To investigate the lipidic composition at the plasma membrane of neutrophils from patients with HTN (n = 6) and normotensive individuals (NTI) (n = 6), we performed quantitative analysis of phospholipids and cholesterol using liquid chromatography coupled with mass spectrometry. Representative chromatograms of phosphatidylcholine (PC), sphingosine (SPH), phosphatidylserine (PS), and phosphatidylethanolamine (PE), along with the concentrations of each phospholipid and cholesterol (CH), are shown in Figure 1A,B. The concentrations of cholesterol and phospholipids were calculated by comparing the peak areas (intensities) with the known mass standards in Figure 1A,B.

**Figure A1:**
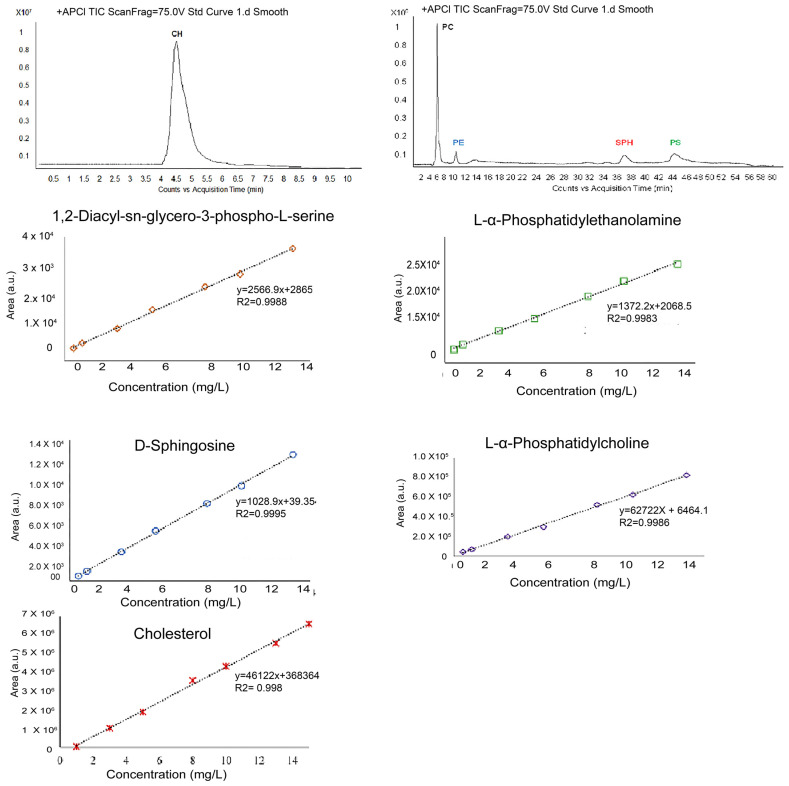
LC-MS/MS Chromatograms and standard curves. Chromatograms and standard curves for phosphatidylserine, phosphatidylethanolamine, sphingosine, phosphatidylcholine, and cholesterol.

Calibration curves and chromatograms were constructed using standards and average peak areas were plotted against the concentrations. Cholesterol was analyzed separately and appeared at 4.5 min (Figure A1). The retention times of the lipids in the neutrophil samples were comparable to the standards; the peaks corresponding to PC, PE, SPH, and PS occurred at 4.5, 7.2, 33.3, and 40.8 min, respectively (Figure A1). Neutrophils from patients with HTN had a higher concentration of cholesterol than neutrophils from NTI 3.4 ± 0.47 vs. 2.83 ± 0.7 μg/10^7^ cells, respectively (*p* = 0.0119; Figure 1A), while the mean ± SE lipid extract concentrations of neutrophils from patients with HTN and NTI were 5.6 ± 1.3 vs. 3.5 ± 0.6 μg/10^7^ cells for PS (*p* < 0.0001), 5.6 ± 1.3 vs. 4.5 ± 0.9 μg/10^7^ cells for PC (*p* = 0.0118), 5.5 ± 1.5 vs. 3.3 ± 0.5 μg/10^7^ cells for PE (*p* = 0.0001), and 5.6 ± 1 vs. 2.8 ± 0.8 μg/10^7^ cells for SPH (*p* = 0.0001), respectively (Figure 1B).

Overall, these results indicate an overexpression of structural phospholipids such as phosphatidylserine (PS), phosphatidylcholine (PC), phosphatidylethanolamine (PE), and sphingomyelin (SPH), and cholesterol (CH).

LC-MS/MS for phosphatidylcholine (PC), sphingosine (SPH), phosphatidylserine (PS), and phosphatidylethanolamine (PE) and a graphical representation of their concentration in the lipid extracts of neutrophils from patients with HTN (red) and NTI (blue). Values are the mean ± standard error (SE) for three biological replicates from six individuals in each group; * *p* = 0.011, ** *p* = 0.012, **** *p* = 0.0001; unpaired *t*-test. HTN: hypertension; NTI: normotensive individuals.

### 2.2. Caveolin-1 Expression Might Alter Internalization of ENaC

Caveolin-1 (Cav1) is the principal component of caveolae and has been reported to inhibit the activity of ENaC by decreasing its expression in epithelial cells [25]. Previously, we suggested that caveolin-1 might regulate the permanence of ENaC at the surface of the plasma membrane of human neutrophils [24].

**Figure 2:**
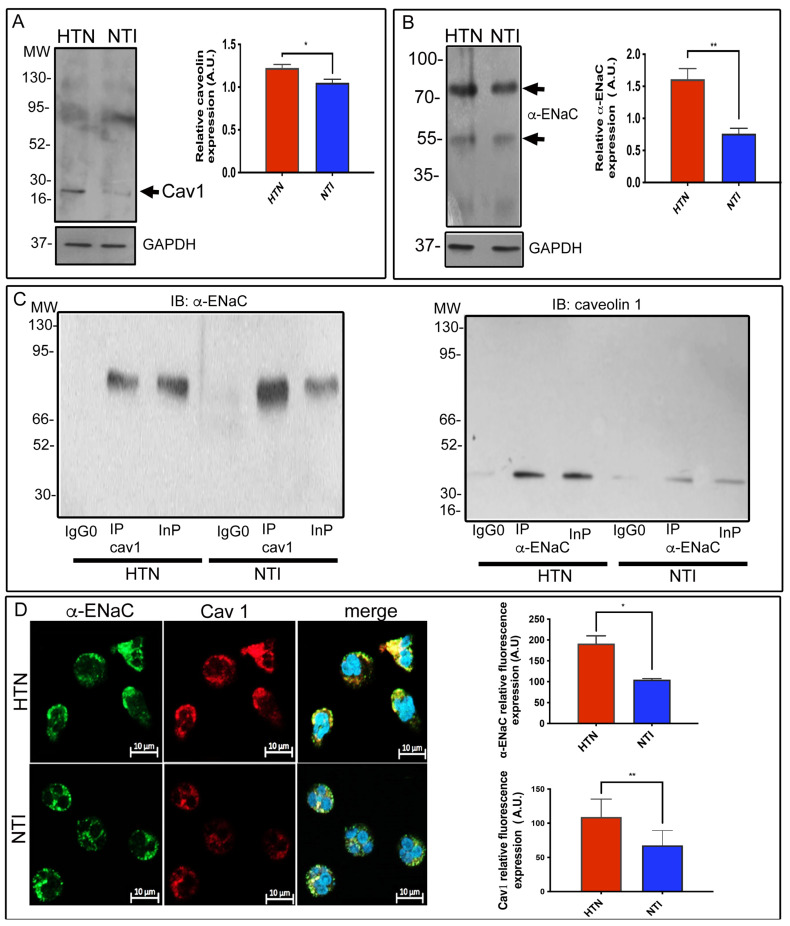
Caveolin-1 is overexpressed in neutrophils from patients with HTN. (**A**) Neutrophils from patients with hypertension (HTN) and normotensive individuals (NTI) were lysed and processed for Western blot assays using an antibody against caveolin-1 (24 kDa). Relative protein expression was quantified using GAPDH as a loading control. Values are the mean ± standard error (SE) for the six individuals in each group. (**B**) Neutrophils from patients with HTN and NTI were lysed and processed for Western blot assays using antibodies against α-ENaC (~75 kDa). Relative protein expression was quantified using GAPDH as a loading control. Values are the mean ± standard error (SE) for the six individuals in each group. ** *p* = 0.0103 for α-ENaC; unpaired *t*-test. (**C**) Neutrophils from patients with HTN and NTI were lysed and processed for immunoprecipitation assays using anti-α-ENaC and anticaveolin-1 antibodies. The total protein extracts (Inp) and immunoprecipitates (IP) were analyzed by immunoblotting; antibodies against α-ENaC detected a band of 75 kDa, and caveolin-1 antibodies detected a band of 24 kDa. No bands were observed using a control antibody (IgG0). (**D**) Neutrophils from patients with HTN and NTI were double-labelled using an α-ENaC antibody detected with Alexa-Fluor-488 and caveolin-1 antibody detected with Alexa-Fluor 568, and fifty cells from three independent experiments were analyzed by confocal microscopy. Nuclei were counterstained with DAPI (4,6-diamidino-2-phenylindole). Scale bar = 10 µm. Graphical representation of the mean ± standard error (SE) relative fluorescence intensities for the six individuals in each group. * *p* = 0.0102 for α-ENaC, and ** *p* = 0.0011 for Cav1; unpaired *t*-test. HTN: hypertension; NTI: normotensive individuals.

In this study, we confirmed that caveolin-1 is expressed at significantly higher relative levels in neutrophils from patients with hypertension (HTN) compared to neutrophils from normotensive individuals (NTI) (1.22 ± 0.071 vs. 1.05 ± 0.068; *p* = 0.0391) with the band detected at ∼24 kDa (Figure 2A). We conducted a confirmation of the overexpression of α-ENaC in neutrophil lysates obtained from six patients with HTN in comparison to neutrophils from six NTI. The elevated expression of α-ENaC in the HTN samples was clearly demonstrated, as indicated by the presence of a distinct band at approximately ∼75 kDa (Figure 2B). Relative quantification demonstrated that the α-ENaC subunit was significantly overexpressed in neutrophils from patients with HTN compared to neutrophils from NTI (1.61 ± 0.288 vs. 0.763 ± 0.142; *p* = 0.0103).

The association between caveolin-1 and α-ENaC was corroborated by immunoprecipitation assays of whole neutrophil lysates using anti-caveolin-1 and anti-α-ENaC, followed by immunoblotting employing the respective antibodies. The anti-caveolin-1 antibody pulled down a band of ∼75 kDa; this same band was observed in the total extracts lane (Inp). Reciprocal experiments performed with the α-ENaC antibody detected caveolin-1 as a band of ∼24 kDa in the Inp and Ip lanes (Figure 2C). No bands were detected in the IgG0 lane.

To assess if there were also differences in the subcellular distribution and expression of caveolin-1 in neutrophils from patients with HTN and NTI, we performed double immunostaining using antibodies raised against α-ENaC and caveolin-1 labeled with Alexa-fluor 488 and Alexa-fluor 594, respectively. Caveolin-1 co-localized with α-ENaC in a uniform punctate pattern around the plasma membrane and cytoplasm of neutrophils from both patients with HTN and NTI. This co-distribution was enhanced and more polarized in the merged images of neutrophils from patients with HTN (Figure 2D). Flourescence relative quantification demonstrated that the α-ENaC and Cav1 were significantly overexpressed in neutrophils from patients with HTN compared to neutrophils from NTI (x = 191.3 ± 18.71 vs. x = 104.9 ± 2.471; *p* = 0. 0102 for α-ENaC and x = 109.1 ± 8.263 vs. x = 67.83 ± 6.853; *p* = 0. 0011 for Cav1), Figure 2D.

**Figure A2:**
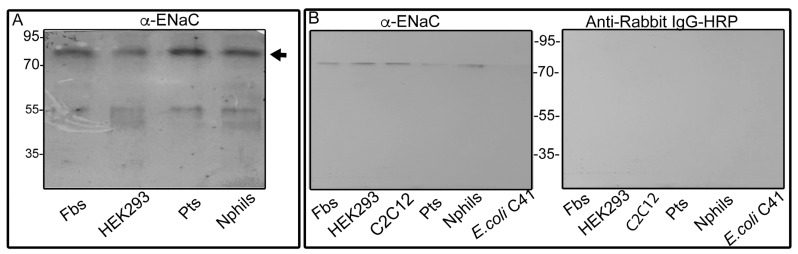
Expression of α-ENaC as a 75 kDa protein. (**A**) Western blot assays were performed on total lysates from fibroblasts (Fbs), Human Embryonic Kidney (HEK-293) cells, platelets (Pts), and neutrophils (Nphils) using an anti-α-ENaC polyclonal antibody, revealing a corresponding band at ~75 kDa (arrow). (**B**) Bands at ~75 kDa were detected in total lysates from fibroblasts (Fbs), Human Embryonic Kidney (HEK-293) cells, C2C12 cells, platelets (Pts), and neutrophils (Nphils). No bands were observed in the protein extracts of *Escherichia coli* C41. No bands were observed with the exposure to the anti-IgG HRP secondary antibody in the absence of primary antibody.

To date, α, β, γ, and δ, subunits have been described and identified in various mammalian cells [26,27]. Previously, we detected the presence of α, ß, and γ-subunits comprising ENaC in neutrophils, with the α-subunit being overexpressed in neutrophils from patients with HTN. We previously reported the overexpression of an α-ENaC isoform of 55 kDa using a discontinued antibody that recognizes a cytoplasmic epitope between residues 20 and 42 of α-ENaC [19]. In the present study, we used an antibody raised against an extracellular domain between residues 365 and 391, which mainly revealed a protein band identified as the α-ENaC isoform of 75 kDa in neutrophils, and although a faint band of 55 kDa is visualized, we only focused on the 75 kDa band, as it represents the known furin cleavage product essential for ENaC activation [28]. We conducted a comparison of total lysates from different cell types that have previously been reported to express α-ENaC, including fibroblasts [29], HEK293 cells [30], and platelets [31], alongside neutrophil lysates. The bands observed in all lysates showed a comigration pattern with a molecular mass of ∼75 kDa, similar to previously reported findings. This confirmation strengthens our confidence in the correct identification of α-ENaC in neutrophils and ensures the reliability of our experimental results (Figure A2A). In addition, we included negative controls using *E. coli* C41 protein extracts as well as anti-rabbit IgG HRP without primary antibody (Figure A2B).

### 2.3. The NEDD4-2/SGK1/ENaC System Is Altered in Neutrophils from Patients with HTN

**Figure 3:**
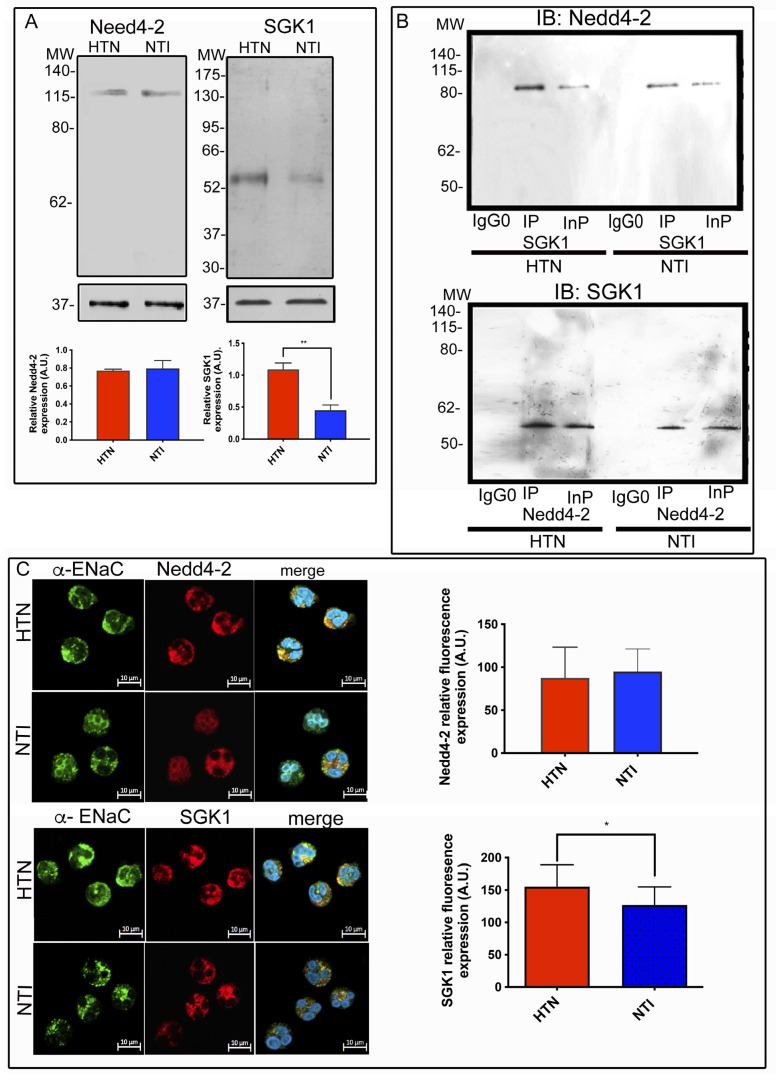
The NEDD4-2-SGK1-ENaC system is altered in neutrophils from patients with HTN. (**A**) Neutrophils from patients with hypertension (HTN) and normotensive individuals (NTI) were lysed and processed for Western blot assays using antibodies against NEDD4-2 (~112 kDa) and SGK1 (~54 kDa). Relative protein expression was quantified using GAPDH as a loading control. Values are the mean ± standard error (SE) for the six individuals in each group. ** *p* = 0.008; unpaired *t*-test. (**B**) Neutrophils from patients with HTN and NTI were subjected to immunoprecipitation assays using anti-NEDD4-2 and anti-SGK1 antibodies (IP). The total protein extracts (Inp) and immunoprecipitates (IP) were analyzed by immunoblotting; antibodies against SGK1 detected a band of ~52 kDa, and NEDD4-2 antibodies detected a band of ~112 kDa. No bands were observed using a control antibody (IgG0). (**C**) Neutrophils from patients with HTN and NTI were double-labeled using an α- ENaC antibody detected with Alexa-Fluor-488 and NEDD4-2 or SGK1 antibodies detected with Alexa-Fluor 568, and fifty cells from three independent experiments were analyzed by confocal microscopy. Nuclei were counterstained with DAPI (4,6-diamidino-2-phenylindole). Scale bar = 10 µm. Graphical representation of the mean ± standard error (SE) relative fluorescence intensities for the six individuals in each group. *p* = 0. 6638 for NEDD4-2, and * *p* = 0.041 for SGK1; unpaired *t*-test. HTN: hypertension; NTI: normotensive.

NEDD4-2 has been recognized as an ENaC-specific ubiquitin ligase and phosphorylation by serum- and glucocorticoid-regulated kinase-1 (SGK1) reduces the ability of SGK1 to bind to ENaC, which increases the presence of ENaC on the plasma membrane [32]. Therefore, to determine whether the NEDD4-2/SGK1/ENaC axis is functional in neutrophils, lysates from neutrophils from patients with HTN and NTI were subjected to Western blotting. NEDD4-2 was observed as a band of ∼112 kDa (Figure 3A), while SGK1 was detected as a band of ∼54 kDa. The relative abundance of NEDD4-2 was similar in neutrophils from patients with HTN and NTI (*p* = 0.793). In contrast, SGK1 was significantly overexpressed in neutrophils from patients with HTN in comparison to neutrophils from NTI (x = 0.449 ± 0.143 vs. x = 1.09 ± 0.173; *p* = 0.008; Figure 3A).

To confirm the association between NEDD4-2 and SGK1, neutrophil protein extracts were subjected to immunoprecipitation assays and the immunoprecipitates (Ip) and total extracts (Inp) were resolved by Western blotting. SGK1 pulled down NEDD4-2 (∼112 kDa), while the complementary immunoprecipitation with NEDD4-2 pulled down SGK1 (∼54 kDa; Figure 3B). No bands were detected in the control immunoprecipitation assay using an irrelevant antibody, confirming the reliability of the assay.

To evaluate the subcellular distribution of NEDD4-2 in relation to ENaC, double immunofluorescent staining and confocal microscopy analysis were performed on neutrophils from patients with HTN and NTI. An abundant patchy polarized pattern of distribution of the α-ENaC subunit was observed at the plasma membrane and in the cytoplasm in neutrophils from patients with HTN, and NEDD4-2 co-localized in the cytoplasmic and plasma membrane zones. In contrast, the α-ENaC subunit exhibited a patchy uniform distribution at the plasma membrane and cytoplasm of neutrophils from NTI, where it co-localized with NEDD4-2 in a discrete way. However, the merged images revealed co-localization of NEDD4-2 with α-ENaC in patches at the plasma membrane in neutrophils from patients with HTN, while fine aggregates of co-localization of NEDD4-2 with α-ENaC were observed at the plasma membrane and in the cytoplasm of neutrophils from NTI (Figure 3C).

The subcellular distribution of SGK1 in relation to α-ENaC was assessed using the respective antibodies and confocal microcopy analysis. SGK1 was observed as intense patches at the plasma membrane and in the cytoplasm and colocalized with α-ENaC in neutrophils from patients with HTN, whereas less intense patches of SGK1 and scarce points of co-localization of SGK1 and α-ENaC were observed in neutrophils from NTI (Figure 3C). Flourescence relative quantification from fifty cells from each group demonstrated that NEDD4-2 has a similar expression in neutrophils from patients with HTN compared to neutrophils from NTI (x = 87.57 ± 13.49 vs. x = 95.03 ± 9.938; *p* = 0. 6638), while SGK1 was significantly overexpressed for NEDD4-2 and (x = 155 ± 10.7 vs. x = 127 ± 8.82; *p* = 0. 0471) for SGK1, Figure 3C.

Taken together, these results demonstrate that SGK1 is overexpressed in the neutrophils of patients with HTN and that there is a close association between NEDD4-2 and SGK1 in neutrophils from both patients with HTN and NTI.

### 2.4. The Ubiquitin Protein Ligase NEDD4-2 Phosphorylation Is Essential to Maintain α-ENaC Expression

**Figure 4:**
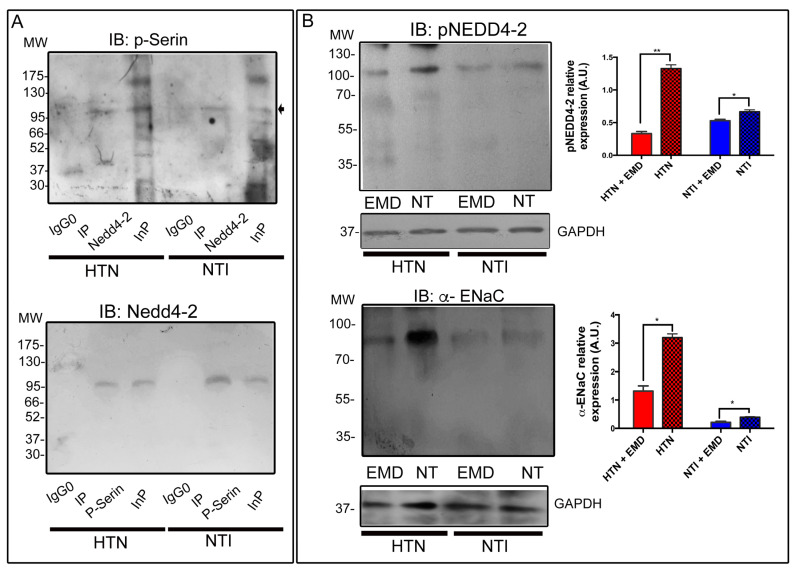
SGK1 escapes from NEDD4-2 regulation. (**A**) Neutrophils from patients with HTN and NTI were lysed and subjected to immunoprecipitation assays using anti-NEDD4-2 and anti-p-serin antibodies or immunoblotting assays using anti-p-serin, anti-NEDD4-2, and anti-p-serin antibodies [IP]. The total protein extracts (Inp) and immunoprecipitates (Ip) were analyzed by immunoblotting utilizing antibodies against NEDD4-2 and p-serin; bands of ~112 kDa were detected. (**B**) Neutrophils from patients with HTN and NTI treated with EMD638683 were lysed and processed for Western blot assays using antibodies against pNEDD4-2 (~112 kDa) and α-ENaC (~75 kDa). Non-treated neutrophils were used as controls. Relative protein expression was quantified using GAPDH as a loading control. Values are the mean ± standard error (SE) for the six individuals in each group. ** *p* = 0.00253 * *p* = 0.0251; RM one-way ANOVA. HTN: hypertension; NTI: normotensive.

Serum- and glucocorticoid-regulated kinase (SGK) phosphorylates Nedd4-2, which targets the epithelial Na^+^ channel (ENaC) for degradation. After establishing that NEDD4-2 and SGK1 are components of the protein complex potentially regulating ENaC in neutrophils, we determined the presence of NEDD4-2 phosphorylated in the serine residue. For this purpose, we immunoprecipitated neutrophil lysates from patients with HTN and NTI with a NEDD-4-2 antibody. Subsequent Western blotting analysis using a P-serin antibody revealed several bands that corresponded to serine-phosphorylated proteins in the input lanes, while a band of ∼112 kDa that might correspond to NEDD4-2 was observed in the lanes containing the immunoprecipitates of neutrophils from patients with HTN or NTI. In the complementary immunoprecipitation using an anti-P-serine antibody, Western blotting using a NEDD4-2 antibody revealed a band of ∼112 kDa corresponding to the molecular weight of NEDD4-2 (Figure 4A). Functional activation of SGK1 requires further phosphorylation, and SGK1, in turn, phosphorylates NEDD4-2, preventing ENaC ubiquitination [33].

To explore whether Sgk1 inhibition diminishes NEDD4-2 phosphorylation (pNEDD4-2) in our studied system, we treated neutrophils from patients with HTN and NTI with the SGK1 inhibitor EMD638683 [34]. Protein lysates of neutrophils from HTN (n = 3) and NTI (n = 3) were processed for Western blotting assays using an antibody raised against phosphorylated NEDD4-2 (pNEDD4-2); GAPDH was used as a loading control. Bands of ∼112 kDa revealed the presence of lower relative quantities of pNEDD4-2 in neutrophils from HTN and NTI treated with EMD638683 compared to non-treated neutrophils from HTN and NTI (Figure 4B; x = 0.331 ± 0.0332 vs. 1.33 ± 0.0578; *p* = 0.0025 and x = 0.0.529 ± 0.0224 vs. 0.667 ± 0.0281; *p* = 0.0251; respectively).

Furthermore, to evaluate the impact of α-ENaC levels in neutrophils from patients with HTN and NTI treated with the SGK1 inhibitor EMD638683, total lysates were processed for Western blotting assays using the α-ENaC antibody. Bands of ∼75 kDa indicated reduced expression of α-ENaC in neutrophils from HTN and NTI treated with EMD638683 compared to non-treated neutrophils from both groups (Figure 4B; x = 1.31 ± 0.185 vs. x = 3.19 ± 0.135; *p* = 0.0106 and x = 0.211 ± 0.0365 vs. x = 0.391 ± 0.0213; *p* = 0.0175; respectively). Our results highlight the key role of SGK in the retrieval and degradation of α-ENaC subunit channels. Phosphorylation of NEDD4-2 reduces its binding to α-ENaC and increases its expression at the cell surface.

### 2.5. Lysosomes Are One via to Clear α-ENaC from Plasma Membrane Neutrophils

**Figure 5:**
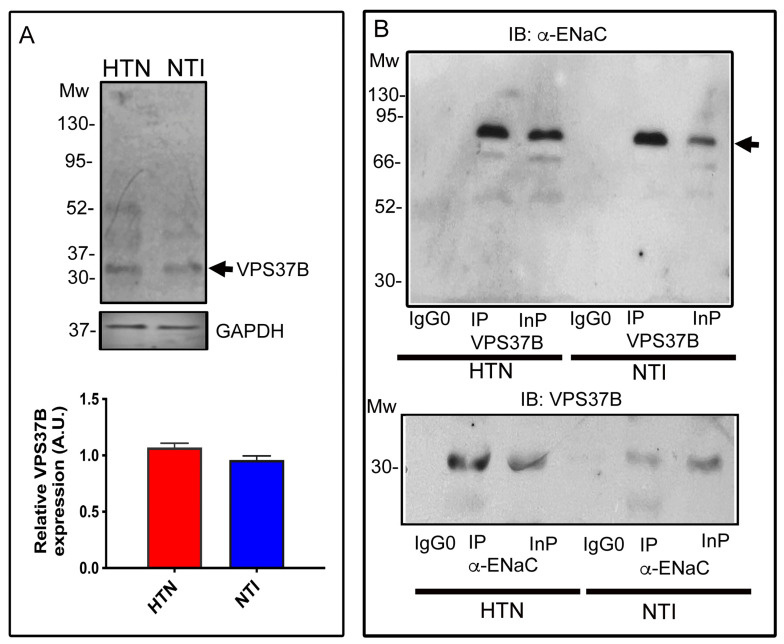
Lysosomes are one avenue to clear α-ENaC from plasma membrane neutrophils. (**A**) Neutrophils from patients with HTN and NTI were lysed and processed for Western blot assays using antibodies against VPS37B (~31 kDa). Relative protein expression was quantified using GAPDH as a loading control. Values are the mean ± standard error (SE) for the six individuals in each group. (**B**) Neutrophils from patients with HTN and NTI were lysed and processed for immunoprecipitation assays using anti-α-ENaC and anti-VPS37B antibodies [IP]. The total protein extracts (Inp) and immunoprecipitates (Ip) were analyzed by immunoblotting; antibodies against α-ENaC detected a band of ~75 kDa, and VPS37B antibodies detected a band of ~31 kDa. No bands were observed using a control antibody (IgG0). HTN: hypertension; NTI: normotensive.

Endosomal sorting of complexes required for transport (ESCRT-I) plays an essential role in sorting ubiquitinated target membrane proteins in the multivesicular body pathway [35,36] before the proteins are trafficked to lysosomes for degradation. ESCRT-I is a heterotetrameric complex that includes vacuolar protein sorting protein 37 (VPS37) [37]. Western blotting for VPS37B detected a band of ∼35 kDa that was expressed at similar levels in neutrophils from patients with HTN and neutrophils from NTI (0.674 ± 0.076 vs. 0.840 ± 0.135; *p* = 0.136; Figure 5A).

The association between VPS37B and α-ENaC was confirmed by immunoprecipitating neutrophil protein extracts with the corresponding antibodies. VPS37b pulled down α-ENaC with a main band of ∼75 kDa and a faint band of ∼55 kDa, while an immunoreactive band corresponding to VPS37b (∼35 kDa) was recovered in the anti-α-ENaC immunoprecipitated fraction. No bands were observed in the control IgG0 lane in these experiments (Figure 5B).

Overall, these results suggest that the ubiquitinated α-ENaC subunit is sorted into multivesicular bodies, which are crucial intermediates in the internalization through the endolysosomal system in neutrophils from HTN and NTI.

### 2.6. ENaC Is Sensitive to Dual Ubiquitination

The pattern of ubiquitination directs ENaC for degradation by different proteolytic pathways: polyubiquitinated ENaC is directed to the proteasome, while mono-ubiquitinated ENaC is degraded by lysosomes [38].

**Figure 6:**
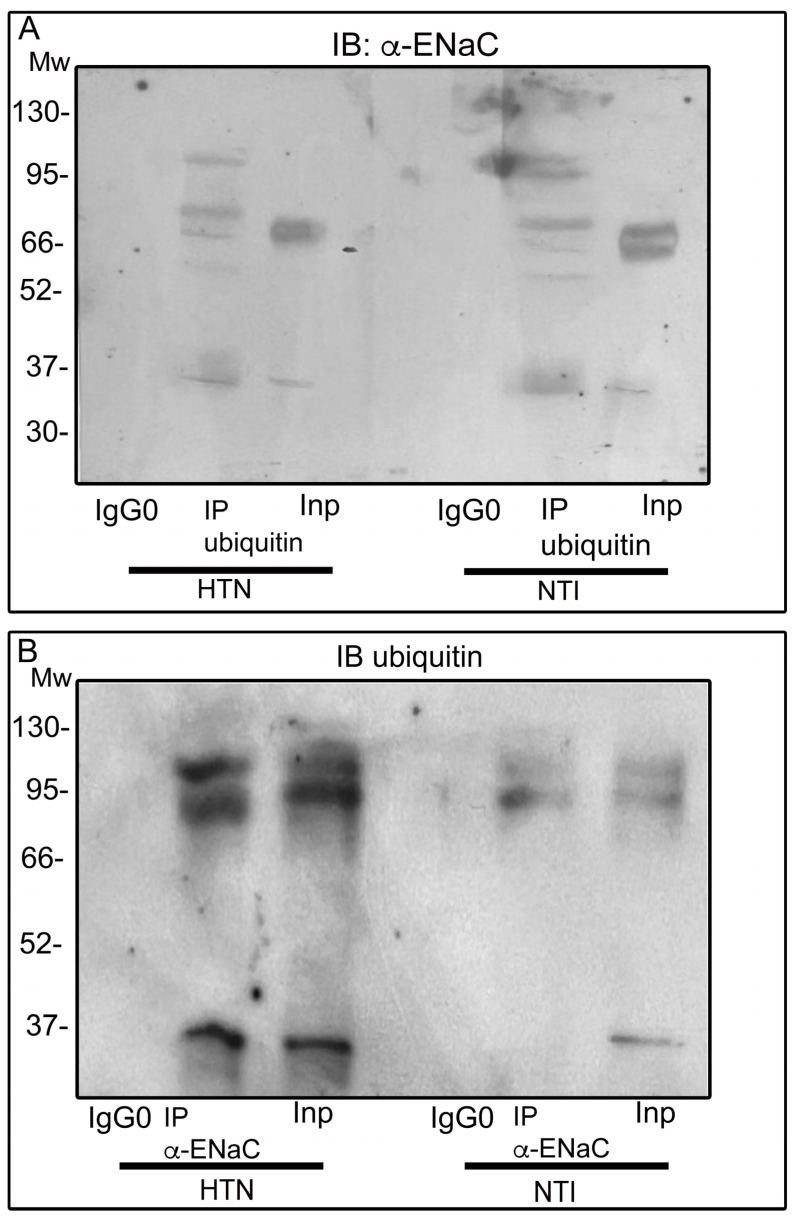
Pattern of ubiquitination of ENaC. Neutrophils from patients with HTN and NTI were subjected to immunoprecipitation assays: (**A**) using anti-ubiquitin and (**B**) anti-α-ENaC antibodies [IP]. The total protein extracts (Inp) and immunoprecipitates (IP) were analyzed by immunoblotting. Antibodies against α-ENaC detected bands of 75 kDa and 85 kDa, and 110 kDa; the ubiquitin antibody detected bands of ~85 kDa, and ~110 kDa. The InP lanes’ α-ENaC detected bands of ~75 kDa; while InP lanes’ ubiquitin detected bands of ~85 kDa and ~110 kDa. No bands were observed using the control antibody (IgG0). HTN: hypertension; NTI: normotensive.

To determine the pattern of ENaC ubiquitination, we immunoprecipitated lysates from neutrophils from three patients with HTN and three NTI using an anti-ubiquitin antibody and performed immunoblotting using an anti-α-ENaC antibody. Figure 6A illustrates the input lanes (InP) of neutrophil lysates obtained from individuals with HTN and NTI; the bands of ~75 kDa, represent the presence of α-ENaC in the protein lysates from neutrophils before the immunoprecipitation procedure. The immunoprecipitation products using anti-ubiquitin antibody (lanes IP ubiquitin) of neutrophil from individuals with HTN and NTI contained bands of ~75 kDa, ~85 kDa, and ~110 kDa corresponding to enrichment of ubiquitinated α-ENaC; a ~100 kDa band was especially enriched in the lysates of neutrophils from NTI (Figure 6A).

Complementary immunoprecipitation assays were performed using anti-α-ENaC, and immunoblotting using an anti-ubiquitin antibody is displayed in Figure 6B. The input lanes (InP) contained several bands corresponding to ubiquitinated proteins (~35 kDa, ~95 kDa, and ~110 kDa), while the IP α-ENaC lane showed bands of ~35 kDa, ~85 kDa, and ~110 kDa, corresponding to the main bands detected in the immunoprecipitations performed using the anti-ubiquitin antibody. These bands were particularly abundant in neutrophil lysates from HTN patients. Ubiquitin was also detected in the control IP as a band of ~9 kDa (Figure A4).

Overall, these results suggest that α-ENaC undergoes both mono- and multi-ubiquitination to a greater extent in neutrophils from patients with HTN than in those from NTI.

### 2.7. ENaC Is Degraded by Two Mechanisms

In order to determine the mechanism(s) responsible for the rapid degradation of ENaC in neutrophils, we tested the effect of inhibitors of either the proteasome system or the endosomal/lysosomal system on the expression of α-ENaC in neutrophils from patients with HTN and NTI.

**Figure 7:**
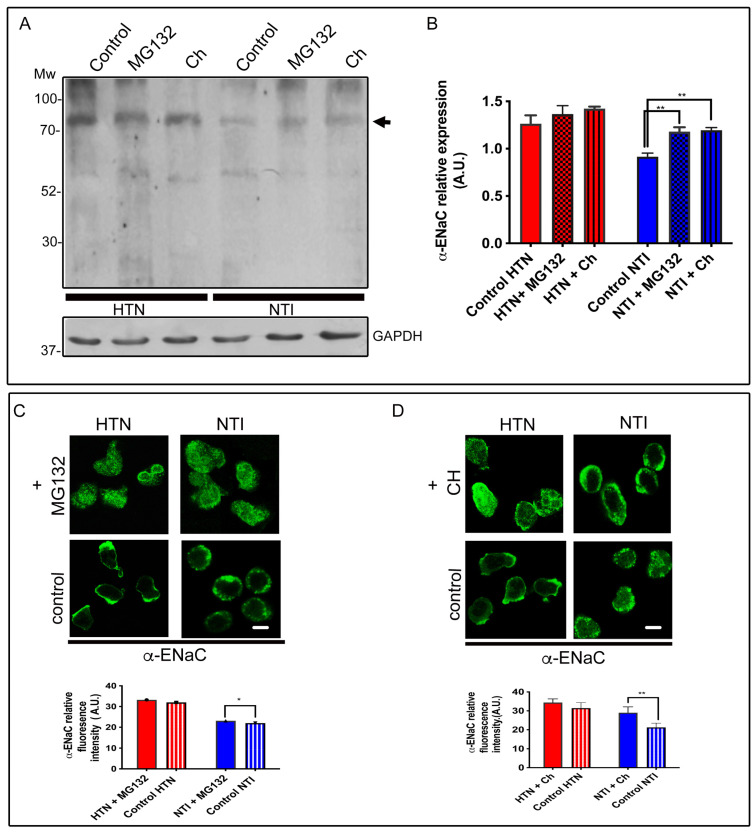
ENaC is degraded by two alternative mechanisms. (**A**) Neutrophils from patients with HTN and NTI were incubated with chloroquine (10 µM) or with MG132 (10 mM), lysed and processed by Western blot assays using an antibody against α-ENaC (~75 kDa). (**B**) Relative protein expression was quantified using GAPDH as a loading control. Values are the mean ± standard error (SE) for the six individuals in each group. ** *p* = 0.0037. (**C**) Neutrophils from patients with HTN and NTI were incubated with MG132 (10 mM) and labeled with α-ENaC antibody detected with Alexa-Fluor-488 and were then analyzed by confocal microscopy. The control treatment was incubated with 0.02% DMSO. Scale bar = 10 µm. Graphical representation of the mean ± standard deviation (SD) of the relative fluorescence intensities for three individuals in each group * *p* = 0.0157. (**D**) Neutrophils from patients with HTN and NTI were incubated with chloroquine (10 µM) and labeled using an α-ENaC antibody detected with Alexa-Fluor-488, and fifty cells from three independent experiments were analyzed by confocal microscopy. The control treatment was incubated with 0.02% DMSO. Nuclei were counterstained with DAPI (4,6-diamidino-2-phenylindole). Scale bar = 10 µm. Graphical representation of the mean ± standard error (SE) of the relative fluorescence intensities for the 10 individuals in each group. ** *p* = 0.0068. HTN: hypertension; NTI: normotensive.

Thus, to confirm this observation, neutrophils from three patients with HTN and six NTI were incubated with 10 mM carbobenzoxy-Leu-Leu-leucinal (MG132) to inhibit the proteolytic activity of the 26S proteasome complex [39] and with 10 µM chloroquine (Ch) to disrupt protein degradation by acidic hydrolases in the lysosome [40]. We conducted Western blotting assays using an α-ENaC antibody (Figure 7A) and performed immunofluorescent double-staining assays using a specific antibody to detect α-ENaC (Figure 7C). The control cells were incubated with 0.02% DMSO.

Treatment with MG132 and chloroquine resulted in an increased relative expression of α-ENaC, detected as a band at ~75 kDa, in neutrophils from both HTN patients and NTI (Figure 7A). Notably, these treatments significantly enhanced α-ENaC expression in neutrophils from NTI compared to treated neutrophils from HTN patients. Specifically, MG132 and chloroquine treatments increased α-ENaC expression in neutrophils from NTI patients compared to NTI controls (x = 0.9152 vs. x = 1.181 ± 0.05416; *p* = 0.0049; and x = 0.9152 vs. x = 1.195 ± 0.05416; *p* = 0.0037; n = 3; respectively; Figure 7B). In contrast, HTN samples treated with MG132 and chloroquine exhibited similar α-ENaC relative expression values (x = 1.264 vs. x = 1.366 ± 0.1043; *p* = 0.5494; x = 1.264 vs. x = 1.429 ± 0.1043; *p* = 0.2849; respectively; Figure 7B)

Confocal analysis of neutrophils from three HTN patients and three NTI treated with MG132 revealed that α-ENaC accumulated as fine patches and was homogeneously distributed throughout the plasma membrane and cytoplasm (Figure 7C). Treatment with chloroquine (Ch) modified the α-ENaC distribution, reinforcing its presence along the plasma membrane with thick patches and less invasion into the cytoplasm (Figure 7D).

These differences were evident when treated neutrophils were compared to controls that were not exposed to any drug. Non-treated neutrophils from HTN individuals showed an elongated morphology characteristic of an activated stage, and α-ENaC was observed with a polarized distribution as previously reported [24], while neutrophils from NTI showed the presence of α-ENaC in a fine patched pattern along the plasma membrane (Figure 7C,D, controls).

Quantitative analysis of immunofluorescence assays of thirty cells from three independent experiments showed that neutrophils from patients with HTN treated with MG132 had similar median α-ENaC fluorescence values to those of HTN control neutrophils (x = 29.44 vs. x = 34.77 ± 3.171; *p* = 0.1438; Figure 7C). This was in contrast to neutrophils from patients with NTI treated with MG132 that exhibited significantly higher median fluorescence values than untreated neutrophils from NTI (x = 20.58 vs. x = 24.67 ± 1.226; *p* = 0.0157; Figure 7C).

Quantification of the fluorescence intensity of thirty cells from three independent experiments demonstrated similar values between neutrophils from HTN treated with chloroquine compared to their untreated controls (x = 34.42 vs. x = 31.46 ± 1.28; *p* = 0.1385), while chloroquine-treated neutrophils from NTI significantly increased α-ENaC expression (x = 29 vs. x = 21.27 ± 1.924; *p* = 0.0068; Figure 7D).

Overall, these results suggest that both the proteasomal and lysosomal systems are ineffective in dealing with the high expression of α-ENaC in neutrophils from patients with hypertension.

## 3. Materials and Methods

### 3.1. Reagents

Unless otherwise indicated, all reagents were purchased from Sigma Chemical Co. (St. Louis, MO, USA), including 1,2-diacyl-sn-3-phospho-L-serine, L-α-phosphatidylcholine, L-α-phosphatidylethanolamine, D-sphingosine, cholesterol, and formic acid (>98%); 0.45 µm nylon membrane filters were purchased from Merck Millipore Ltd. (Carrigtwohill, Cork, Ireland). All solvents used for analytical determinations were HPLC grade. Table 1 lists the antibodies used in this study.

### 3.2. Patients and Ethical Approval

Blood samples (15 mL) were isolated from six patients with HTN and six normotensive individuals (NTI), and the sera were processed immediately to perform clinical determinations (glucose, triglycerides, and cholesterol). Patients with diabetes mellitus or dyslipidemia were excluded from this study. All patients enrolled in the study had well-controlled arterial tension values and their blood pressure was monitored weekly by a healthcare professional before and after taking the blood samples.

The anthropometric data of the individuals included in the HTN and NTI groups are presented in Table 2. The HTN group had mean blood pressure values of 138/89 mmHg; the NTI group had mean blood pressure values of 117/79 mmHg and no personal or familial history of HTN. The mean age and body weight (BW) of both groups were similar. Patients with HTN were previously diagnosed with essential hypertension and were receiving treatment that included angiotensin-converting enzyme (ACE) inhibitors (such as enalapril), angiotensin II receptor antagonists (losartan, telmisartan), or a calcium channel blocker (amlodipine; Table 3).

This study was approved by the Bioethical Committee of ENMyH Instituto Politécnico Nacional (No. 001/2019). All patients provided signed informed consent for the procedure to be carried out as well as for their participation in the study and the publication of the results obtained.

### 3.3. Neutrophil Isolation

Neutrophils were isolated from 15 mL of peripheral blood in the presence of ethylenediaminetetraacetic acid (final concentration, 4 mM) and were separated using a density separation medium, as described previously [24]. In brief, 10 mL of blood was centrifuged at 1000× *g* for 20 min at 10 °C in the presence of cold Histopaque 1.077 g/mL separation gradient. The pellet, containing both red blood cells and neutrophils, was washed with Hank’s balanced salt solution (HBSS) without calcium (137 mM NaCl, 5.3 mM KCl, 1 mM MgCl_2_, 0.28 mM Na_2_HPO_4_.12H_2_O, 0.87 mM NaH_2_PO_4_, 0.44 mM KH_2_PO_4_, 4.1 mM NaHCO_3_, 5.5 mM glucose) and centrifuged for 5 min at 450× *g*. Red blood cells were lysed in 1 mL of distilled water for 30 s, mixed with 1 mL of 1.8% salt solution, and centrifuged for 5 min at 450× *g*. The isolated neutrophils were resuspended in HBSS.

### 3.4. Cell Culture

Primary human dermal fibroblasts (AG08469 Coriell), HEK293 cells (CRL1573 ATCC), and C2C12 (CRL1772 ATCC) were cultured in MEM, DMEM/F-12, and DMEM, respectively (Invitrogen, Carlsbad, CA, USA). The culture media were supplemented with 15% (*v*/*v*) fetal bovine serum (Biowest, Nuaillé, France), 50 U/mL penicillin, and 50 μg/mL streptomycin (Sigma-Aldrich, St. Louis, MO, USA). Cells were maintained at 37 °C in a humidified atmosphere containing 5% CO_2_. Platelets were obtained by venipuncture from human donors following the procedure described by Cerecedo et al. [31].

### 3.5. Preparation of Inhibitors

A 10 µM chloroquine solution (2x) (Sigma Chemical Co., St. Louis, MO, USA), a 10 mM MG132 solution (2x) (Sigma Chemical Co., St. Louis, MO, USA), and a 25 µM EMD638683 solution (2x) (Cayman Chemical Co., Ann Arbor, MI, USA) were prepared in PBS (137 mM NaCl, 2.7 mM KCl, 8 mM Na_2_HPO_4_, and 2 mM KH_2_PO_4_) from concentrated solutions diluted in dimethyl sulfoxide (DMSO) to achieve a final DMSO concentration of 0.02%. Indeed, the additional effects of proteasome inhibitors on cell cycle progression and induction of apoptosis are well-known. However, the concentration and duration of incubation we used had the desired effect of inhibiting the chymotrypsin-like activity of the proteasome [41]. 

### 3.6. Treatment of Neutrophils with Inhibitors

Neutrophils in suspension were incubated with the same volume of the drugs to obtain the previously mentioned final concentrations of chloroquine 10 µM, MG132 10 mM, and EMD63868325 µM for 60 min at room temperature.

### 3.7. Western Blotting

Neutrophil lysates from patients with HTN and NTI were resuspended in sample buffer, boiled for 5 min, subjected to 10% SDS-polyacrylamide gel electrophoresis (PAGE), and transferred onto nitrocellulose membranes using a semi-dry system (Thermo Electron Co., Milford, MA, USA). Membranes were incubated with appropriate primary antibodies (Table 1 followed by horseradish peroxidase (HRP)-conjugated secondary antibodies, and the bands were visualized using an enhanced-chemiluminescence Western-blotting analysis system (Santa Cruz Biotechnology, Inc., Santa Cruz, CA, USA) and documented using G/RA film (Kodak, Rochester, NY, USA). Negative control blots were incubated solely with HRP-conjugated secondary antibodies. Densitometry analysis was performed using Win Image Studio Digits ver. 4.0 software (LI-COR, Inc., City, NE, USA); GAPDH was used as a loading control to normalize the data.

### 3.8. Immunoprecipitation Assays

Neutrophils from patients with HTN and NTI were lysed for 15 min at 4 °C in an equal volume of 2× lysis buffer (2 mM EGTA, 100 mM HEPES, 150 mM NaCl, and 2% NP40, pH 7.4) containing protease inhibitor cocktail. The lysates were incubated for 2 h at 4 °C with the immunoprecipitating antibodies and subsequently incubated overnight with A/G agarose beads (Santa Cruz). Immunoprecipitates (IP) were separated by centrifugation, washed with NP40-free lysis buffer and then re-suspended in 2× sample buffer (125 mM Tris–HCl, 4% SDS, 20% glycerol, 0.01 mg/mL β-mercaptoethanol and bromophenol blue, pH 6.8) and boiled for 5 min. Immunoprecipitated proteins (IP) and inputs were resolved by Western blotting. An antibody against GFP raised in rabbit and an antibody against GST raised in mouse were used as controls and referred to as the irrelevant antibody according to the nature of the immunoprecipitating antibody.

### 3.9. Immunofluorescent Staining

**Figure A3:**
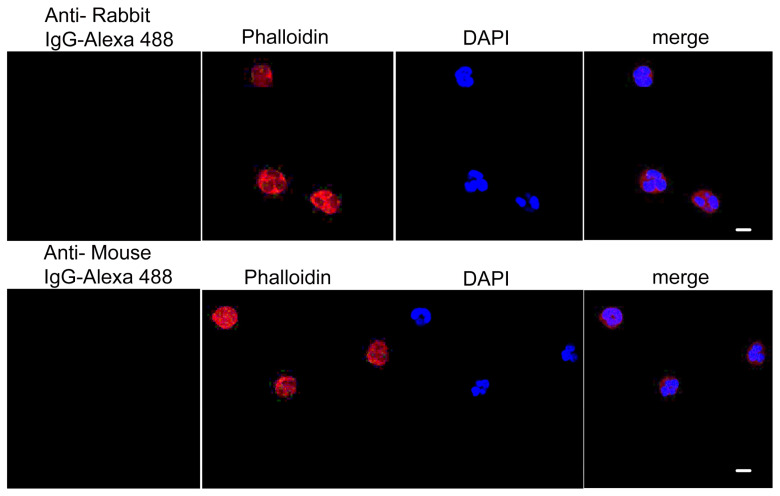
No fluorescence signal was observed in neutrophils that were double-labeled using an anti-rabbit IgG-Alexa 488 and an anti-mouse IgG-Alexa 488. Cells were also stained with phalloidin, and nuclei were counterstained with DAPI (4,6-diamidino-2-phenylindole). Scale bar = 10 µm.

**Figure A4:**
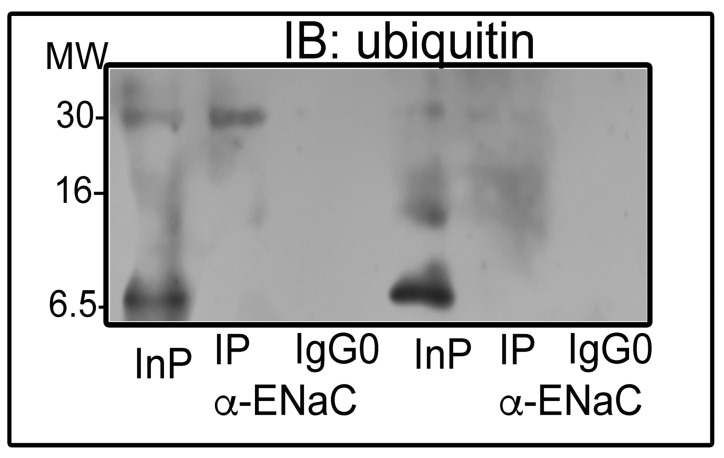
Ubiquitin control. Immunoprecipitation (IP) using an anti-α-ENaC antibody revealed bands of approximately 8 kDa in the Inp lanes corroborate the immunoprecipitation of ubiquitin. No bands were observed using a control antibody (IgG0).

Neutrophils from patients with HTN and NTI were seeded onto poly-D-lysine-coated coverslips, allowed to adhere for 60 min, permeabilized and fixed with a mixture of 2% p-formaldehyde and 0.2% Triton X-100, incubated with the specific primary antibodies (Table 1) diluted in 0.1% BSA in PBS for 2 h, washed with PBS, and then incubated for 1 h with secondary antibodies conjugated to Alexa-Fluor-488 or Alexa-Fluor 594 (Molecular Probes, Eugene, OR, USA); nuclei were counterstained with 4′,6-diamidino-2-phenylindole (DAPI). Actin filaments were identified using tetramethyl rhodamine iso-thiocyanate (TRITC)-phalloidin. The slides were washed, mounted, and observed using a Zeiss LSM-800 confocal laser scanning microscope (TCP-SP5, Leica, Heidelberg, Germany) employing a Plan Neo Fluor 63Å~(NA = 1.4) oil immersion objective. Negative controls included cells incubated only with an anti-rabbit IgG-Alexa 488 and an anti-mouse IgG-Alexa 488. Neutrophils were stained with tetramethyl rhodamine iso-thiocyanate (TRITC)-phalloidin to show the presence of cells; nuclei were counterstained with 4′,6-diamidino-2-phenylindole (DAPI) (Figure A3).

### 3.10. Quantitative Analysis of Phospholipids and Cholesterol

Neutrophils from patients with HTN and NTI (1 × 10^7^) were lysed in 100 μL of 2× lysis buffer (0.5% NP-40, 2 mM Na_3_VO_4_, PSMF) in the presence of protease inhibitor cocktail, diluted to 1000 μL with PBS, and processed for lipid extraction, as previously described [42]. In brief, neutrophil suspensions were extracted in 3 mL of chloroform:methanol (2:1, *v*:*v*) by vortexing for 2 min, centrifuged for 5 min at 2000× *g*, and the chloroform fraction was evaporated to dryness under reduced pressure at room temperature using a BÜCHI R-215 Rotary Evaporator coupled to a BÜCHI V-700 Vacuum Pump and cold-water recirculating BÜCHI Chiller F-105 (BÜCHI Labortechnik AG, Flawil, Switzerland). The dried extracts were diluted in 1 mL of methanol for analysis.

### 3.11. Analysis and Quantification of Phospholipids and Cholesterol

Lipid analysis was performed in triplicate for all samples via liquid chromatography coupled to triple quadrupole mass spectrometry (LC-MS/MS). Chromatographic separation of 1,2-diacyl-sn-3-phospho-L-serine (PS), L-α-phosphatidylcholine (PC), L-α-phosphatidylethanolamine (PE), and D-sphingosine (SPH) was achieved using two serial reverse-phase capillary columns. Briefly, a 5 µL sample was injected into the LC device and passed through a Zorbax Eclipse Plus C18 column (2.1 mm × 150 mm; 3.5 µm particle size), followed by a SB-C18 column (4.6 mm × 100 mm, 5 μm particle size). The temperature was 25 °C for both columns. The mobile phase was composed of a mixture of methanol (solvent A) and acetonitrile (solvent B); the flowrate was 0.4 mL min^−1^. Initially, 20% of solvent A was provided for 5 min, increased to 90% of solvent A by 60 min, returned to 20% of solvent A at 65 min, and maintained for 5 min. Analytes were identified and quantified using a triple quadrupole mass spectrometer (Agilent Technologies, Inc., Santa Clara, CA, USA) in positive electrospray ionization mode (ESI+) for the first fragmentation (Table S1) and single ion-monitoring (SIM) mode for quantitative analysis. The calibration curves for the analytes are shown in Figure 1A.

For cholesterol, a 20 µL sample was injected and separated on a Zorbax SB-C18 (4.6 mm × 100 mm, 5 μm particle size) capillary column. The mobile phase consisted of an isocratic mixture of acetonitrile:methanol (80:20) at 0.4 mL/min^−1^. Mass analysis was conducted using atmospheric-pressure chemical ionization in positive mode (APCI+); the ions produced by APCI were analyzed with the triple quadrupole device using the conditions indicated in Table S1. The quality of the analysis was validated using an external standard (Sigma-Aldrich).

### 3.12. Statistical Analysis

Statistical analysis was carried out with GraphPad Prism^®^ statistical software Version 10.1.0 (264) (GraphPad Software, Inc., La Jolla, CA, USA). Unpaired *t*-tests were employed for data analysis, and the results are presented as mean values with standard error (±SE). Statistical significance was defined as *p* < 0.05.

## 4. Discussion

The present study represents the first comprehensive investigation of the relationship between the structural lipid environment of the plasma membrane and associated proteins involved in the retrieval and degradation of ENaC in neutrophils. In this study, we conducted lipid analysis using whole cells rather than characterizing structural lipids from isolated plasma membrane of neutrophils. However, in physiological and pathological conditions, changes in lipid composition mainly occur at the cell membrane. These changes can affect the biophysical properties of microdomains that might be associated with a variety of diseases, including cancer, obesity, neurodegenerative disorders, and cardiovascular pathologies, among others [43].

We demonstrated an increase in the expression of cholesterol (CH), phosphatidylserine (PS), phosphatidylcholine (PC), phosphatidylethanolamine (PE), and sphingosine (SPH) in neutrophils from hypertensive (HTN) patients compared to neutrophils from normotensive individuals (NTI).

It is feasible that the changes in the membrane phospholipid bilayer might not only alter the membrane biophysical properties [24] of neutrophils from hypertensive individuals but also influence the selection of the mode of internalization of ENaC [44,45].

According to the evidence observed in the present study, we suggest that cholesterol may impact the high expression of ENaC at the plasma membrane of neutrophils from hypertensive (HTN) patients in two different ways. First, at the outer zone of the plasma membranes, cholesterol, in association with sphingolipids and caveolin-1, forms part of lipid rafts [46]. This association might promote the high density of ENaC, as sphingolipids and caveolin-1 were highly expressed. Second, through the inner leaflet of the plasma membrane, cholesterol regulates ENaC activity by interacting with PIP2 [47,48]. Therefore, it would be interesting for future studies to evaluate ENaC activity, as the reduction of cholesterol might decrease its activity, while the increased expression of PS might maintain ENaC activity [49].

The interdependence of cholesterol in promoting ENaC activation by cytosolic SGK1 [20] was evident in neutrophils from hypertensive (HTN) patients. It is feasible that SGK1, caveolin-1, and cholesterol promote the permanence of ENaC at the plasma membrane, as the presence of an intact lipid environment and/or lipid rafts as a signaling platform are needed to regulate ENaC ubiquitination. Caveolin-1 participates in the interaction with ENaC and Nedd4-2 to internalize ENaC and induce its ubiquitination; however, the overexpression of caveolin-1 might also promote an abundance of ENaC at the plasma membrane [25].

Neutrophils from hypertensive patients exhibit morphological and biochemical similarities to activated neutrophils [24]. These features suggest that the alterations in the levels of phosphatidylcholine (PC), phosphatidylserine (PS), and phosphatidylethanolamine (PE) correspond to the activated state of circulating neutrophils in hypertensive patients [50,51,52]. The notably high concentration of sphingosine (SPH), a foundational component of sphingolipids, in hypertensive neutrophils aligns with elevated SGK1 levels, a known promoter of ceramide and sphingolipid biosynthesis [53], which in turn might favor the presence of ENaC at the plasma membrane of hypertensive neutrophils. From the evidence presented to date, the most potent downregulation of ENaC is initiated by the ubiquitination of the channel and retrieval from the apical membrane by endocytosis [19]. We postulated that the increased expression of the α-subunit of ENaC in hypertensive patient neutrophils might be linked to alterations in its endocytic trafficking at the plasma membrane, potentially directing ENaC toward degradation pathways.

Initially, we assessed the role of the ubiquitin protein ligase NEDD4-2, a known regulator of ENaC abundance and function in the kidney and colon [54]; the unphosphorylated form of NEDD4-2 targets ENaC for degradation, while the phosphorylated form targets SGK1 [11,55]. The expression of NEDD4-2 between neutrophils from patients with HTN and NTI was similar, and the phosphorylation of NEDD4-2 at a serine position 488 (Figure 4A) may prevent ubiquitination of ENaC in both neutrophils from HTN patients and NTI. Phosphorylated NEDD4-2 favors the ubiquitination of SGK1 in kidney cells [56], and in accordance with our results, negative feedback between NEDD4-2 and SGK1 might be present in both neutrophils from patients with HTN and NTI (Figure 4B). The use of EMD 638683, an inhibitor of SGK1 [34], demonstrated that SGK1 is the primary kinase responsible for regulating the phosphorylation of ENaC and may modulate its activity. In addition, the inhibition of SGK and the prevalence of NEDD4-2 phosphorylated at lower levels suggest that the function of ENaC may also be regulated by other ubiquitin protein-ligases such as Cbl-l [57]. In addition, the phosphorylation of NEDD4-2 by a specific kinase or its regulation through transcriptional control mechanisms remains a viable possibility. Moreover, it is essential to consider the heightened levels of calcium within the cytoplasm of neutrophils from hypertensive patients [58], potentially inducing the translocation of ENaC to the cell surface [59].

ENaC is a heteromeric channel consisting of three homologous subunits: α, β, and γ [60]. Additionally, a δ-ENaC subunit has been identified, which preferentially assembles with β and γ subunits to form a δβγ-channel. This δβγ-ENaC configuration has been detected in various cell types, including lymphocytes [60,61], and exhibits biochemical and pharmacological differences when compared to αβγ-ENaC [62]. Given the distinctive properties between these ENaC configurations, our laboratory has initiated an investigation into the expression of δβγ-ENaC in neutrophils from hypertensive individuals.

In addition, we determined that α-ENaC might be monoubiquitinated and polyubiquitinated, directing its transport for degradation in both the proteasome and the lysosome, respectively, in a similar manner in neutrophils from hypertensive (HTN) patients and controls. However, we suggest that both degradation processes are insufficient to counter the overexpression of α-ENaC.

Antihypertensive medications, such as thiazide diuretics and aldosterone antagonists, are known to modulate the activity of the epithelial sodium channel (ENaC). In non-epithelial cells, there is a strong relationship between aldosterone and ENaC expression, similar to that observed in the renal epithelium. However, the exact mechanisms of aldosterone effects on neutrophils remain unclear [63,64].

Our results support the hypothesis that neutrophils play a role in hypertension development; however, we cannot yet establish the clinical impact of targeting ENaC in neutrophils as a pharmacologically alternative treatment against hypertension.

## 5. Conclusions

This study indicates that alterations in the lipid composition of the plasma membrane in neutrophils from HTN patients could affect the structure of microdomains where ENaC is located, potentially impacting its endocytosis. Ubiquitin conjugation plays a pivotal role in the degradation of ENaC in both HTN and NTI patient neutrophils, with NEDD4-2 acting as ubiquitin ligase for the α-ENaC subunit. Additionally, we found that the overexpression of SGK1 also contributes to maintaining ENaC at the plasma membrane, and neither lysosome nor proteasome degradation mechanisms are sufficient to counter the overexpression of ENaC in HTN patient neutrophils. We recognize that this study is a first approximation, focusing on the expression of certain proteins that might be involved in ENaC degradation. However, other experimental techniques must be considered to fully explain the high expression of α-ENaC at the plasma membrane in neutrophils from hypertensive patients. Understanding the regulation or dysregulation of factors responsible for the retrieval of ENaC may contribute to a better understanding of the pathogenesis of hypertension. Such insights may also have significant implications for the development of targeted therapies aimed at modulating SGK1 activity or enhancing ENaC degradation to effectively manage and treat hypertension.

## Figures and Tables

**Table 1:** Characteristics of the antibodies used.

Antibody	Catalog Num	Specificity	Dilution Wb/IF/IP	Resource
anti-VPS37B	SC-390144	monoclonal	WB 1:100 IF 1:20	Santa Cruz Biotechnology (Santa Cruz, CA, USA)
anti-CIN85	SC-166862	monoclonal	WB 1:100 IF 1:20	Santa Cruz Biotechnology (Santa Cruz, CA, USA)
anti-SGK	SC-28338	monoclonal	WB 1:100 IF 1:20	Santa Cruz Biotechnology (Santa Cruz, CA, USA)
anti-caveolin 1	SC-53564	monoclonal	IF 1/60	Santa Cruz Biotechnology (Santa Cruz, CA, USA)
anti-Ubiquitin	SC-8017	monoclonal	WB 1/500 IP 2 µg	Santa Cruz Biotechnology (Santa Cruz, CA, USA)
anti-caveolin 1	SC-101653	polyclonal	WB 1:100	Santa Cruz Biotechnology (Santa Cruz, CA, USA)
anti-GAPDH	SC-32233	polyclonal	WB 1:500	Santa Cruz Biotechnology (Santa Cruz, CA, USA)
anti-α-ENaC	TA3250073	polyclonal	WB 1:1000 IF 1:20 IP 2 µg	OriGene Technologies Inc. (Rockville, MD, USA)
anti-Nedd4-2	GTX130730	polyclonal	WB 1:150 IF 1:20 IP 2 µg	Genetex (Irvine, CA, USA)
anti-pNedd4-2	TA379036	polyclonal	WB 1:250	OriGene Technologies Inc (Rockville, MD, USA)
anti-P-serine	SC-81514	monoclonal	WB 1:500 IP 2 µg	Santa Cruz Biotechnology (Santa Cruz, CA, USA)
Anti-GFP	SC-8334	polyclonal	IP 2 µg	Santa Cruz Biotechnology (Santa Cruz, CA, USA)
Anti-GST	SC-138	Monoclonal	IP 2 µg	Santa Cruz Biotechnology (Santa Cruz, CA, USA)

**Table 2:** General characteristics of hypertensive patients and controls.

Parameter	Hypertensive	Controls	*p* Value
Sex (male/female)	2/4	3/3	---------
Age ± SD (years)	60 ± 9.26	45 ± 12.56	0.2439
BMI	28 ± 5	27 ± 4	0.0898 ns
SBP (mmHg)	138 ± 23	117 ± 5	0.0019
DBP (mmHg)	89 ± 8	79 ± 4	<0.0001

Values are the mean ± SD of 6 patients and 6 controls in each group. BMI, body mass index; SBP, systolic blood pressure; DBP, dystolic blood pressure; ns, non-significant.

**Table 3:** Number of individuals using each antihypertensive medication class (%).

ACEI	1 (16.6)
ARB	3 (50)
BB	1 (16.6)
CCB	1 (16.6)

CEI = angiotensin-converting enzyme inhibitors, ARB = angiotensin II receptor blockers, BB = beta blockers, CCB = calcium channel blockers.

## Data Availability

Supporting reported results are available upon request.

## Supplementary Materials

The following supporting information can be downloaded at: https://www.mdpi.com/article/10.3390/ijms25094939/s1.
