# Supplementary material for: Altered Plasma Membrane Lipid Composition in Hypertensive Neutrophils Impacts Epithelial Sodium Channel (ENaC) Endocytosis
Source: Int J Mol Sci. 2024 Apr 30;25(9):4939. doi: 10.3390/ijms25094939 (PMC11084340; doi:10.3390/ijms25094939)
Supplement: Supplementary file 1 [file ijms-25-04939-s001.zip › ijms-2961794-supplementary.pdf]

Table S1. Mass spectrometry conditions used in the identification and quantification of the biomolecules in neutrophils extract samples

Analyte	Followed ions in SIM mode (m/z)	Drying gas temperature (ºC)	Drying gas flow (L min^{-1})	Capillary voltage (V)	Fragmentator (V)
1,2-diacyl-sn-3-phospho-L-serine	790.4	300	11	4000	135
L-α-phosphatidylcholine	760.5				
L-α-phosphatidylethanolamine	745.4				
D-sphingosine	300.2				
Cholesterol	369.3	325	4	500	135
